# Imaging of neuroinflammation due to repetitive head injury in currently active kickboxers

**DOI:** 10.1007/s00259-022-05715-x

**Published:** 2022-02-15

**Authors:** Gilles N. Stormezand, Janine Doorduin, Sandra E. Rakers, Jacoba M. Spikman, Joukje van der Naalt, David Vállez García, Anouk van der Hoorn, Chris W. J. van der Weijden, Berry P. H. Kremer, Remco J. Renken, Rudi A. J. O. Dierckx

**Affiliations:** 1 Department of Nuclear Medicine and Molecular Imaging, Medical Imaging Center, University of Groningen, University Medical Center Groningen, Hanzeplein 1, 9700 RB Groningen, The Netherlands; 2 Department of Clinical Neuropsychology, University of Groningen, University Medical Center Groningen, Groningen, The Netherlands; 3 Department of Neurology, University of Groningen, University Medical Center Groningen, Groningen, The Netherlands; 4 Department of Radiology, Medical Imaging Center, University of Groningen, University Medical Center Groningen, Groningen, The Netherlands; 5 Cognitive Neuroscience Center, University Medical Center Groningen, University of Groningen, Groningen, The Netherlands

**Keywords:** Repetitive head injury, Neuroinflammation, Kickboxers, TSPO PET

## Abstract

**Purpose:**

Chronic traumatic encephalopathy refers to a neurodegenerative disease resulting from repetitive head injury of participants in contact sports. Similar to other neurodegenerative diseases, neuroinflammation is thought to play a role in the onset and progression of the disease. Limited knowledge is available regarding the neuroinflammatory consequences of repetitive head injury in currently active contact sports athletes. PET imaging of the 18-kDa translocator protein (TSPO) allows quantification of microglial activation in vivo, a marker of neuroinflammation.

**Methods:**

Eleven rank A kickboxers and 11 age-matched controls underwent TSPO PET using [^11^C]-PK11195, anatomical MRI, diffusion tensor imaging, and neuropsychological testing. Relevant imaging parameters were derived and correlated with the outcomes of the neuropsychological testing.

**Results:**

On a group level, no statistically significant differences were detected in non-displaceable binding potential (*BP*_ND_) using PET. Individually, 3 kickboxers showed increased *BP*_ND_s in widespread regions of the brain without a correlation with other modalities. Increased FA was observed in the superior corona radiata bilaterally. DTI parameters in other regions did not differ between groups.

**Conclusion:**

Despite negative results on a group level, individual results suggest that neuroinflammation may be present as a consequence of repetitive head injury in active kickboxers. Future studies using a longitudinal design may determine whether the observed TSPO upregulation is related to the future development of neuropsychiatric symptoms.

**Supplementary Information:**

The online version contains supplementary material available at 10.1007/s00259-022-05715-x.

## Introduction

Chronic traumatic encephalopathy (CTE) refers to a neurodegenerative disease resulting from repetitive head injury in participants of contact sports [1]. It can manifest with neuropsychiatric symptoms which may be affective (depression), behavioral (aggression), and/or cognitive (concentration, memory, or executive impairments). If these latter symptoms are severe, this can be qualified as dementia. The clinical presentation is correlated with different pathological stages (I–IV) [2]. Contact sporters with the APOE epsilon-4 allele have been shown to have an increased risk of developing CTE, and the severity of symptoms is positively linked to the cumulative dose of head impacts [3]. Although neuropsychiatric consequences of repetitive head injury have been described as early as in 1928 by Martland in boxers, it later became apparent that the disease could also affect rugby players, American football players [4], wrestlers [5], or not be related to sports [6]. Typically, the onset of the clinical syndrome is after the athletes have ended their careers [7].

Currently, a definite diagnosis of CTE can only be established post mortem. In 2015, consensus criteria became available which defined accumulation of abnormal tau proteins in neurons and astroglia around small blood vessels at the depth of cortical sulci in an irregular pattern as pathognomonic for the disease [8]. In a series of 202 former American football players, Mez and colleagues found neuropathological evidence of CTE in 177 individuals, with a prevalence of up to 99% in former National Football League (NFL) players [9], the highest professional level. Recently, diagnostic criteria have become available for the clinical diagnosis during life [10]. Although not yet part of the diagnostic criteria, neuroimaging may be a valuable modality in CTE. Tau imaging using [^8^F]-Flortaucipir PET has shown promise to serve as a potential biomarker for CTE in vivo, although it may lack sensitivity in the early stages of the disease [11]. Aspecific features such as a degree of cortical atrophy (especially frontal lobes), ventricular enlargement, or increased prevalence of a cavum septum pellicidum may be revealed by anatomical imaging in the latter stages of the disease [12, 13].

Considerable debate exists as to what is driving the neurodegenerative disease and what is causing the latency in symptom onset [14]. In traumatic brain injury (TBI), neuroinflammation is thought to play an important role [15, 16]. Neuroinflammation refers to an inflammatory response in the brain which can involve the activation of microglia and astrocytes. This can be beneficial when, for example, pathogens are eliminated from the brain, such as in the case of stroke. However, in case of neurodegenerative diseases, microglia activation may become chronic and have a deleterious effect on the brain and thus on cognitive functioning of patients. This detrimental process, coined “immuno-excitotoxicty” [17], is thought to be triggered by repetitive head injury (RPI) resulting in axonal degeneration and microtubular degeneration [17, 18]. In currently active athletes, cerebrospinal fluid (CSF) analysis of the triggering receptor on myeloid cells 2 (sTREM2) has been used as a means to detect activated microglia in vivo [19]. Disadvantages of using CSF as a biomarker of activated microglia are that it lacks information on the spatial distribution of activated microglia and that it requires a lumbar punction. From this perspective, neuroimaging may be a suitable less invasive alternative.

18-kDa translocator protein (TSPO) PET is the most widely studied research modality to image neuroinflammation in vivo. Increased expression of TSPO is mediated by activated microglia and reactive astrocites [20]. In TBI, TSPO PET has shown evidence of widespread microglial activation, which can persist even after 6 months [21]. TSPO imaging may be a valuable tool to assess the severity and location of TBI [22]. It has also been used in other types of CNS insults such as stroke. In stroke, TSPO PET was used to investigate the inflammatory response to ischemia in the core infarction region, peri-infarct zone, and in the contralateral hemisphere [23]. Increased expression of TSPO in currently active or recently retired athletes using PET was first described by Coughlin et al. in 2017 in NFL players (14 subjects and 16 controls) [24]. Since then, no additional reports have appeared assessing TSPO expression using PET in currently active athletes, particularly not in other contact sports. In this proof of principle study, we aim to assess the presence of neuroinflammation using [^11^C]-PK11195 PET, a first-generation TSPO PET tracer, in competitive kickboxers. Kickboxing is a combat sport involving the use of punches and kicks with fists, knees, and elbows. Head injuries represent nearly half of injuries incurred by professional Muay-Thai boxers [25]. Competitive kickboxers are thus exposed to cumulative repeated injury to the head. Eleven rank A kickboxers and eleven controls underwent [^11^C]PK11195 PET/CT scanning and MR imaging, whereas neuropsychological testing was performed to correlate and interpret imaging findings.

## Methods

### Participants

Competitive kickboxers with rank A and a minimum of 20 competitive bouts and 3 training sessions per week were recruited from gyms in the Netherlands. Rank A is the highest rank in kickboxing allowing participation in full contact bouts and can only be achieved after several years of intensive training. Kickboxers were not allowed to have fought a recent match or sustained a recent concussion (within 3 months of the scan). Healthy, age-matched controls without a history of TBI or participation in contact sports were recruited with local advertisements. All participants denied the use of anti-inflammatory drugs or anabolic steroids.

All subjects were asked to fill out two questionnaires: a quality of life scale (SF-36) [26], which is routinely used in the follow-up of mild TBI by the Department of Neurology of the University Medical Center Groningen, and the hospital anxiety and depression scale [27]. In addition, information was gathered in relation to sociodemography, medication, use of alcohol and cigarettes, disease history, history of mild TBI, and fighting record (wins–losses-draws). All subjects underwent neuropsychological testing, with tests for the following domains: intelligence (Dutch version of the national adult reading test [28]), mental speed and attention (trail making test (TMT) A and TMT B [29], Vienna test system (VTS) [30], the 15 words test [31] (both immediate and delayed recall (IR, DR)), digit span test [32], executive functioning (zoo map, subtest of the behavioral assessment of the dysexecutive syndrome (BADS [33]), Hayling test [34], controlled oral word association test (COWAT [35]), and emotion recognition (FEEST [36]). Laboratory testing included serum C-reactive protein (CRP) and interleukine-6 (IL-6) as peripheral markers of inflammation.

### Radiochemistry

[^11^C]-PK11195 was labeled by trapping ^11^C-methyl iodide in a solution of 1 mg of (*R*)-*N*-desmethyl-PK11195 and 10 mg of potassium hydroxide in 300 μL of dimethylsulfoxide. The reaction mixture was allowed to react for 1 min at 40 °C, neutralized with 1 mol/L HCl, and passed through a 45-μm Millex-HV filter (Millipore). The filtrate was purified by high-performance liquid chromatography (HPLC) using a μBondapak C18 column (7.8 × 300 mm) (Waters) with acetonitrile/25 mM NaH_2_PO_4_ (pH 3.5; 55/45) as the eluent (flow, 5 mL/min). To remove the organic solvents from the product, the collected HPLC fraction (retention time, 7 min) was diluted with 100 mL of water and passed through an Oasis HLB 30-mg (1 mL) cartridge. The cartridge was washed twice with 10 mL of water and subsequently eluted with 1 mL of ethanol and 8 mL of water. The product was sterilized by filtration over a 0.20-μm Millex-LG filter (Millipore). Quality control was performed by HPLC, using a Novapak C18 column (150 × 3.9 mm) (Waters) with acetonitrile/25 mM NaH_2_PO_4_ (pH 3.5) (60/40) as the eluent at a flow of 1 mL/min. The radiochemical purity was always greater than 95%. No differences were found between the net injected dose in healthy volunteers (333 ± 53 MBq) and that in patients (336 ± 27 MBq) (*p* = 0.679).

### Imaging

Subjects fasted for 6 h prior to the PET scan. [^11^C]-PK11195 was injected with a speed of 0.5 mL/s followed by a 60-min dynamic PET scan consisting of 23 frames with increasing length on a Siemens Biograph mCT system (Siemens Medical Solutions USA, Inc.) with the head immobilized in a headrest to reduce motion artifacts. Images were reconstructed using Truex + TOF with 3 iterations and 21 subsets in a 400 × 400 matrix size (zoom 1.0). After intravenous injection of [^11^C]-PK11195, arterial blood radioactivity was continuously monitored with an automated sampling system. In addition, 5 extra blood samples were collected at 10, 20, 30, 45, and 60 min after [^11^C]-PK11195 injection to determine the amount of radioactivity in blood and plasma for calibration of the blood monitor, and to determine the amount of radioactive metabolites in plasma. MRI-T1w images were acquired for PET co-registration purposes. Subjects were scanned at a Philips Intera 3.0 T MRI scanner (Philips, Best, the Netherlands) with a 32-channel head coil. A 3D T1w TFE image was acquired for each subject using the following parameters: 160 sagittal slices without gap, FOV (ap × rl × fh) 256 × 160 × 256 mm, acquired matrix 256 × 256, voxel size 1 × 1 × 1 mm, repetition time 9 ms, echo time 3.5 ms, and flip angle 8 degrees. Diffusion-weighted imaging (DWI) was performed using a single-shot spin-echo echo-planar imaging sequence with the following parameters: 65 axial slices of 2 mm without gap, FOV 240 × 240 × 130 mm^3^, acquisition matrix 117 × 120, reconstructed voxel size 1.88 × 1.88 × 2 mm^3^, TR 8877 ms, TE 60 ms, flip angle 90°, 60 diffusion directions (and seven non-diffusion-weighted scans averaged to one volume), and *b*-value 0 and 1000 s/mm^2^. The noise of the diffusion scans was first reduced [37, 38] and corrected for Gibbs ringing artefacts [39]. This was followed by motion correction with correction for eddy-current induced distortions [40, 41] and bias field correction [42]. Subsequently, the diffusion data were subjected to diffusion tensor analysis using FMRIB Software Library (FSL v6.0.4.) [43].

All PET images were coregistered to the individual anatomical T1w MRI and spatially normalized to Montreal Neurological Institute (MNI) space using PMOD (version 4.0, PMOD Technologies Ltd, Zürich, Switzerland). Brain regions were defined using the Hammers maximum probability atlas (Hammers N3083) containing frontal lobes, parietal lobes, temporal lobes (with exception of the amygdala and the hippocampus, which were analyzed separately), occipital lobes, cerebellum, striatum, and white matter. In addition a whole brain grey matter (GM) region was generated, combining all cortical regions. For each patient, the binding potential (*BP*_ND_) was calculated, defined as *k*_*3*_*/k*_*4*_, and used as outcome measure, using a 2 tissue compartment model with *K*_*1*_/*K*_*2*_ fixed to the value obtained for whole brain GM. For kinetic modeling, frame length-dependent weighting was used for fitting the time activity curve, a correction for blood delay was used, and blood data was fitted [44]. Fixing of *k*_*4*_ to the whole brain GM value was performed in subregions when this parameter could not be adequately calculated (e.g., in case of a very high standard error (> 25%), occurring in 7 subjects). Group level comparisons were performed using independent sample Mann–Whitney U tests, with statistical threshold for significance set at 0.05. A non-parametric test was chosen because it was anticipated that a non-normal distribution could be present in kickboxers with individual outliers in [11]C-PK11195 binding depending on the RPI exposure. For statistical analysis, SPSS was used (IBM Corp. Released 2015. IBM SPSS Statistics for Windows, Version 23.0. Armonk, NY: IBM Corp). Correlations between regional *BP*_ND_ and the scores on the neuropsychological testing and questionnaires were assessed using scatter plots and Spearman’s rank correlation coefficients using GraphPad Prism version 7.02 for Windows (GraphPad Software, La Jolla California USA, www.graphpad.com).

**Table 1:** Subject characteristics

	Boxers (*n* = 11)	Controls (*n* = 11)	*p* value (*t*-test)
Mean age (years)	31.1 (6.5)	26.6 (6.1)	0.10
Sex (male, %)	100%	90%	0.34
SF 36:			
- Physical functioning	99.5 (1.6)	99 (2.1)	0.56
- Role limitation (physical)	90 (31.6)	92.5 (23.7)	0.84
- Bodily pain	83 (22.9)	90.5 (16.7)	0.42
- General health	83.9 (11.9)	83.5 (14.9)	0.95
- Vitality	76.0 (11.3)	71.0 (11.5)	0.34
- Social functioning	91.4 (15.6)	85.3 (14.1)	0.37
- Role limitation (mental)	100 (0)	79.9 (35.9)	0.09
- Health changes	55.0 (10.5)	57.5 (12.1)	0.63
- Mental health	80.4 (10.1)	77.6 (11.5)	0.57
HADS:			
- Anxiety	3.8 (2.4)	5.0 (2.7)	0.28
- depression	2.5 (2.5)	3.1 (1.8)	0.84
Peripheral inflammatory markers:			
- IL-6 (pg/ml)	0.9 (0.8)	1.4 (0.8)	0.12
- CRP (mg/L)	0.6 (0.3)	2.1 (4.9)	0.32
Education*	5.3 (0.9)	5.7 (0.8)	0.29
Smoking (*n*)	2	2	1.00
Bouts fought	38.4 (24.4)	-	-
- Won	27.3 (20.3)		
- Lost	9.5 (5.4)		
- Drawn	4.9 (1.2)		
Years in training	15.8 (7.1)	-	-
Frequency of training (per week)	4.9 (1.2)	-	-
Time since last bout (months)	15.8 (15.2)	-	-
Comorbidities	PTSS (n = 1)	-	

Data are displayed in means with the standard deviation. *(**1** less than 6 grades’ primary education, **2** 6 grades’ primary education, **3** 8 grades’ primary education, **4** vocational secondary education, **5** lower general secondary school, **6** higher general secondary school, pre-university school, **7** university)

**Fig. 1:**
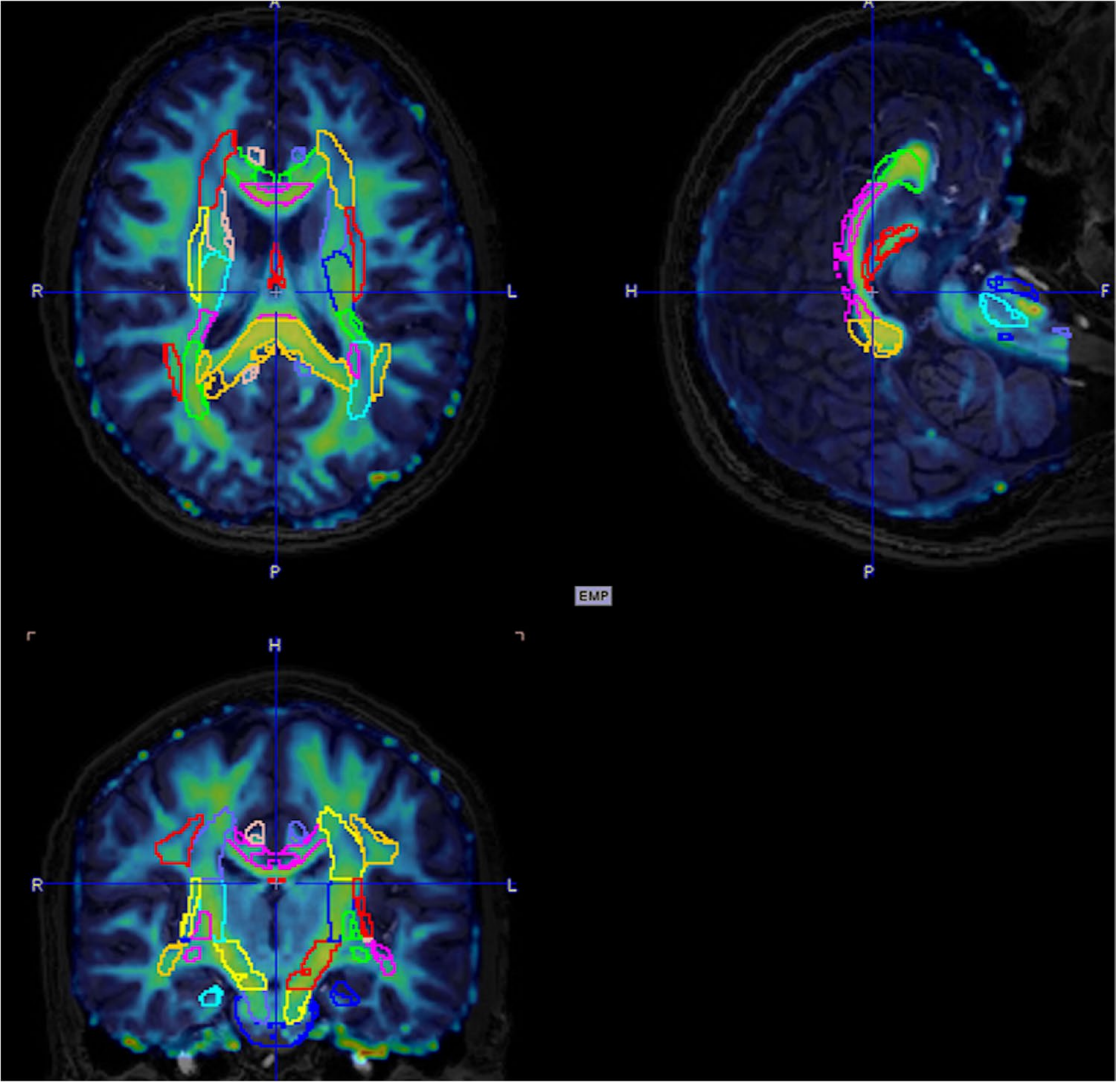
Fused FA/3D T1w MRI image after normalizing to atlas space. The VOIs delineate the white matter regions included in the DTI analyses

Diffusion tensor imaging data were generated with FSL. The fractional anisotropy (FA) and mean diffusivity (MD) maps were spatially normalized to the ICBM-DTI-81 maximum probability maps [45] using PMOD. Atlas-based white matter regions were automatically defined. FA and MD values of predefined white matter regions (Fig. 1, corpus callosum (genu, body, splenium), anterior limb of internal capsule, posterior limb of internal capsule, retrolenticular part of the internal capsule, anterior corona radiata, superior corona radiata, posterior corona radiata, posterior thalamic radiation, superior longitudinal fasciculus) were measured. Similar to previous DTI studies in sports related repetitive head injury, these white matter regions were selected in order to be sensitive to potential changes related to projections from widespread areas of the brain, especially long axonal projections [24]. Additional MRI sequences included SWI, T2w flair, and DTI. All MRI images were visually assessed by a neuroradiologist (AvdH) for the presence of structural abnormalities**.**

## Results

Thirteen kickboxers and 13 age-matched controls were included in the study. Due to technical problems related to the PET scanning procedure (PET reconstruction problem (*n* = 1), failure to place arterial lining (*n* = 3)), 11 kickboxers and 11 controls remained for the final analysis. Subject characteristics are shown in Table 1. The mean age was 31.1 ± 1.9 for the kickboxers and 26.6 ± 1.8 years for the control group (*p* = 0.10). No significant differences were observed in the SF-36 scores, education level, or inflammatory markers in serum. One kickboxer reported a posttraumatic stress disorder. Otherwise, no (psychiatric) comorbidities were present in both kickboxers and controls. One kickboxer had a history of participating in mixed martial arts. Regarding neuropsychological testing (Table 2), kickboxers displayed significantly faster reaction times in the VTS (RT-S1-DT, RT-S2-DT, RT-S3-DR). These are computerized determination tests which study attentional capacity and reaction speed among continuously changing acoustic and visual stimuli. Kickboxers performed worse on the TMT-B. The TMT is a test of executive functioning, and under the B condition, numbered as well as lettered circles have to be connected. Lower scores reflect lower cognitive flexibility [46]. No significant differences between subjects and controls in other tests were detected, although kickboxers tended to perform worse on the 15 word memory test.

**Table 2:** Results of neuropsychological tests

	Boxers (*n* = 11)	Controls (*n* = 11)	*F*	*p* value (ANOVA)
Estimated premorbid IQ	99.0 (2.58)	102.4 (1.77)	1.13	0.30
15 word test:				
- Instant recall	47.2 (2.42)	53.7 (2.17)	4.06	0.06
- Delayed recall	10.64 (0.62)	12.09 (0.39)	3.91	0.06
Digit span:				
- CR forward	9.82 (0.57)	9.18 (0.64)	0.55	0.47
- CR backward	7.55 (0.37)	7.27 (0.80)	0.10	0.76
VTS:				
-RT-S1-DT	238.1 (10.12)	278.1 (10.52)	7.51	0.01
-RT-S2-DT	197.0 (6.63)	233.3 (9.34)	10.03	0.005
-RT-S3-DR	325.1 (12.81)	394.8 (13.52)	14.02	0.001
-DT-S1-correct	235.1 (6.31)	241.9 (6.35)	0.58	0.46
TMT-A	27.60 (4.32)	26.18 (3.00)	0.08	0.79
TMT-B	71.0 (8.65)	50.36 (3.91)	4.72	0.04
Zoo map (total score)	11.09 (1.86)	13.27 (0.82)	1.15	0.30
Hayling (overall scaled)	6.40 (0.34)	5.91 (0.37)	0.95	0.34
COWAT	43.18 (4.42)	44.73 (3.54)	0.07	0.79
FEEST	49.09 (1.56)	49.45 (1.34)	0.03	0.86

Data are displayed in means with the standard error of the mean

**Fig. 2:**
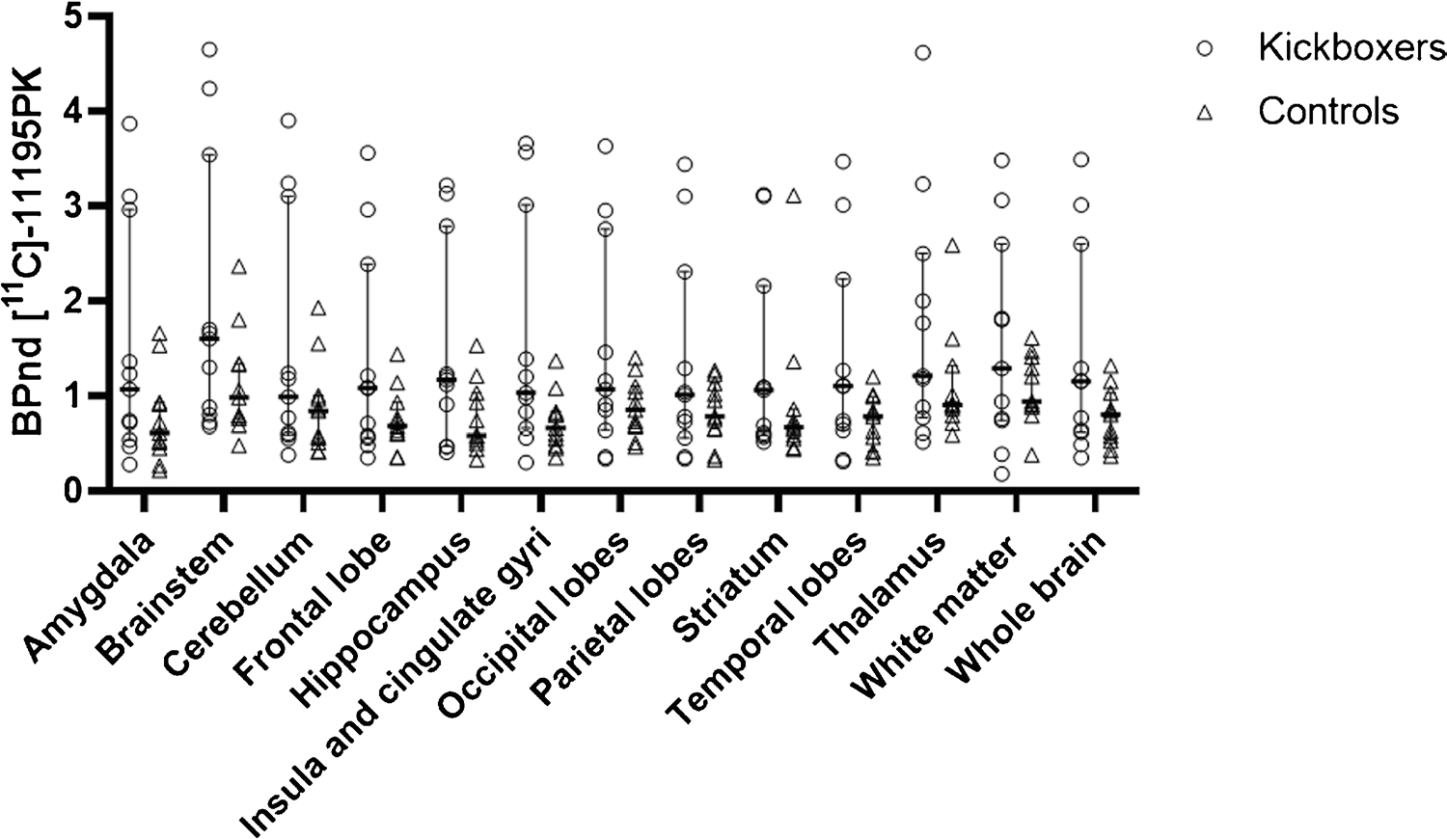
*BP*nd’ of [^11^C]-PK11195 in kickboxers (*n* = 11) and controls (*n* = 11). The symbols represent individual subjects. The horizontal bar reflects the median and the error bars the 1st and 3rd quartile

**Fig. 3:**
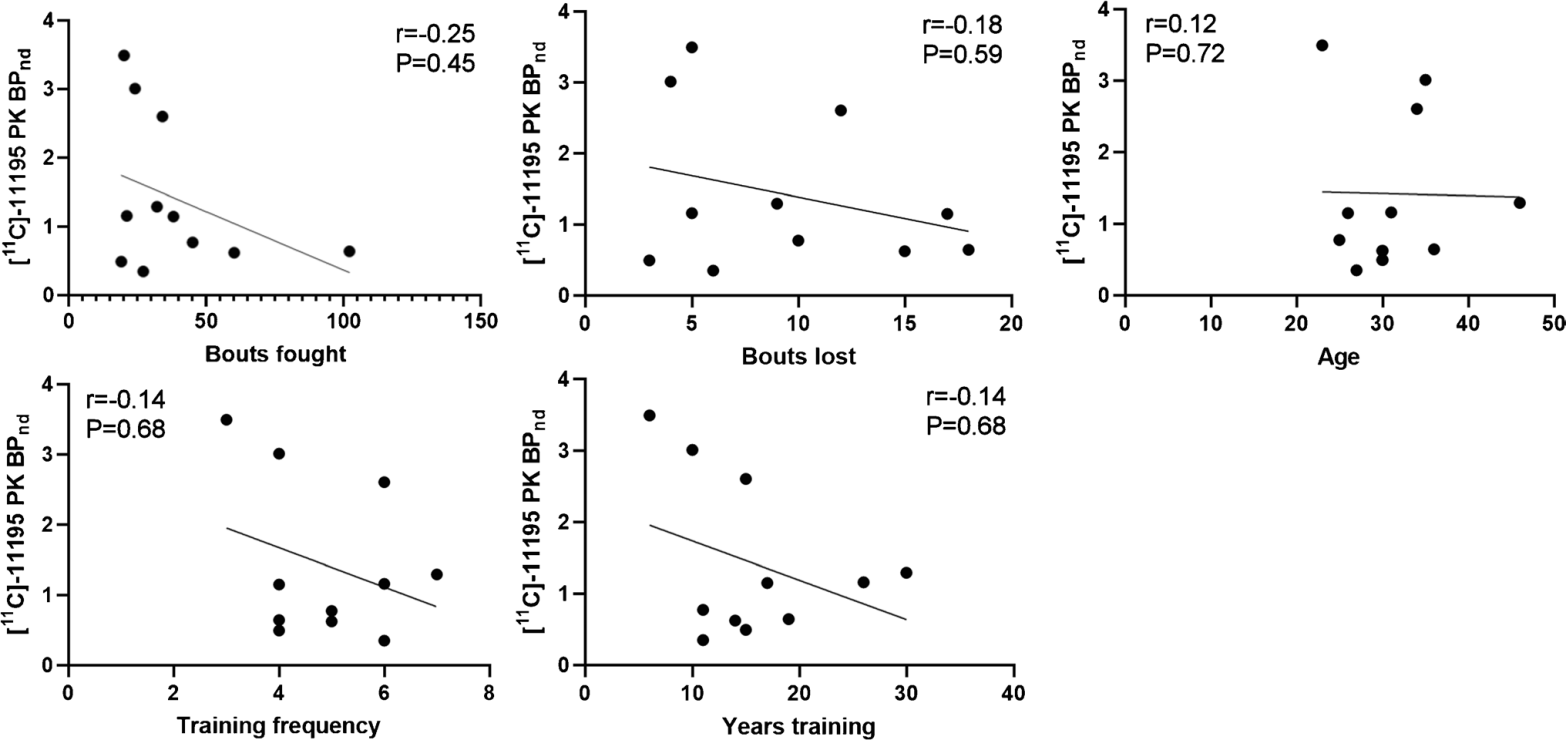
Scatter plots showing the relation between the whole brain BPnds of [^11^C]-PK11195 and bouts fought, bouts lost, age, training frequency, and years training. Spearman’s rank correlation coefficient r is displayed in each graph

**Fig. 4:**
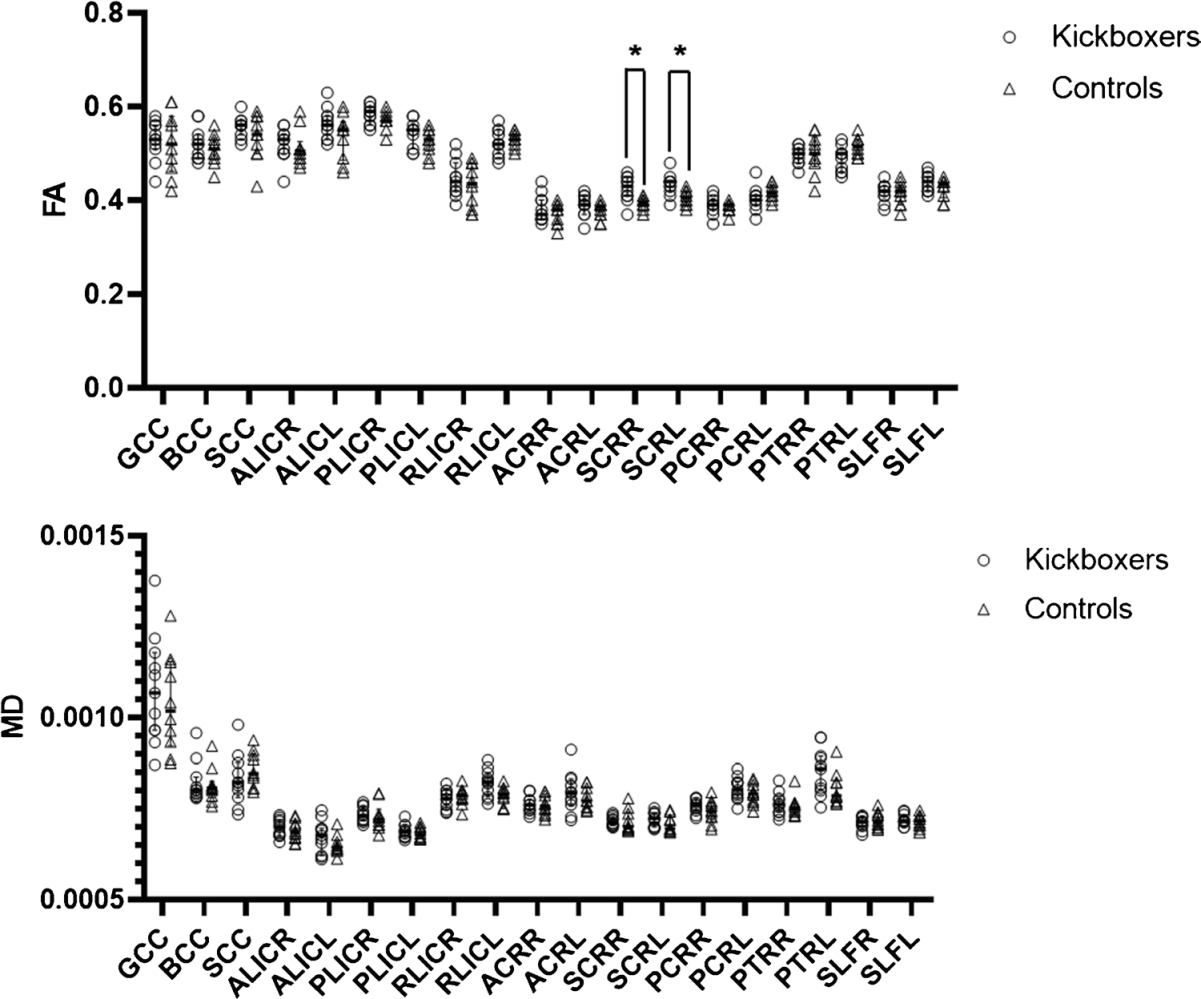
FA and MD values of kickboxers and controls in white matter regions. The symbols represent individual subjects. The horizontal bar reflects the median and the error bars the 1st and 3rd quartile. **GCC** genu corpus callosum, **BCC** body corpus callosum, **SCC** splenium corpus callosum, **ALIC R** anterior limb of internal capsule right, **ALIC L** anterior limb of internal capsule left, **PLIC R** posterior limb of internal capsule right, **PLIC L** posterior limb of internal capsule left, **RIC R** retrolenticular part of internal capsule right, **RIC L** retrolenticular part of internal capsule left, **ACR R** anterior corona radiata right, **ACR L** anterior corona radiata left, **SCR R** superior corona radiata right, **SCR L** superior corona radiata left, **PCR R** posterior corona radiata right, **PCR L** posterior corona radiata left, **PTL R** posterior thalamic radiation right, **PTR L** posterior thalamic radiation left, **SLF R** superior longitudinal fasciculus right, **SLF L** superior longitudinal fasciculus left. *Significant difference

On a group level, no statistically significant differences were found between the *BP*_ND_s in kickboxers and healthy controls (Table 3). Other relevant parameters of the analysis (*K*_*1*_*, K*_*1*_/*K*_*2*_) are shown in the supplemental data (Table S4, Fig. S5). Individually, three kickboxers consequently showed high *BP*_ND_s in nearly all regions evaluated, with the highest *BP*_ND_s being observed in the brainstem and the thalamus (Fig. 2). Without these outliers, medians were similar in both groups (*BP*nd whole brain 0.71 in kickboxers vs. 0.80 in controls, *p* = 0.90). Mean whole brain *k3* and *k4* were 0.21 and 0.06 in the kickboxers with high *BP*nd vs. 0.02 and 0.03 in those with normal *BP*nd (*p* = 0.21 and 0.44, *T*-test). There was no correlation between the measured *BP*_ND_s of [^11^C]-PK11195 in the whole brain and age, bouts fought, bouts lost, years training, and training frequency in kickboxers (Fig. 3). Significantly increased FA values were observed in the superior corona radiata (SCR) bilaterally in kickboxers compared with control subjects (SCR right 0.42 vs. 0.39, *p* = 0.002, left 0.44 vs. 0.41, *p* = 0.006). The measured FA and MD values in other white matters regions did not differ significantly between groups (Fig. 4, Table S5, supplemental data). One control showed a susceptibility artifact, interpreted as a microbleed, whereas none was present in the group of kickboxers. None of the participants showed T2 flair white matter abnormalities. In one kickboxer, a cavernous malformation was detected as an incidental finding. The three kickboxers with high WB *BP*_nd_s did not achieve scores below the 5th or higher than the 95th percentile on neuropsychological testing. No significant correlations were observed between the results on neuropsychological testing and regional *BP*_ND_s in kickboxers.

**Table 3:** Median binding potential (*BP*_ND_ and interquartile ranges) of [^11^C]-PK11195 PET in kickboxers and controls

Brain region	Kickboxers (*n* = 11)	Controls (*n* = 11)	*p* value (Mann–Whitney U)	Effect size r
Brainstem	1.60 (0.80–3.54)	0.98 (0.76–1.34)	0.22	0.39
Frontal lobes	1.08 (0.56–2.39)	0.68 (0.60–0.93)	0.40	0.26
Hippocampus	1.17 (0.47–2.79)	0.58 (0.50–1.03)	0.17	0.43
Amygdala	1.07 (0.54–2.96)	0.61 (0.45–0.93)	0.13	0.46
Temporal lobes	1.10 (0.64–2.23)	0.78 (0.42–0.99)	0.24	0.37
Parietal lobes	1.01 (0.66–3.01)	0.78 (0.65–1.13)	0.44	0.24
Occipital lobes	1.07 (0.64–2.76)	0.85 (0.68–1.10)	0.37	0.28
Insula and cingulate gyri	1.03 (0.66–3.01)	0.66 (0.48–0.83)	0.06	0.58
Thalamus	1.21 (0.77–2.50)	0.90 (0.79–1.32)	0.37	0.29
Striatum	1.06 (0.57–2.16)	0.67 (0.55–0.86)	0.22	0.37
Cerebellum	0.99 (0.59–3.10)	0.84 (0.51–1.00)	0.30	0.33
White matter	1.29 (0.74–2.60)	0.94 (0.88–1.41)	0.52	0.21
Whole brain	1.15 (0.62–2.60)	0.80 (0.53–1.03)	0.24	0.36

## Discussion

In this study, no significantly increased whole brain *BP*_ND_s of [^11^C]-PK11195 were observed in a group of highly competitive kickboxers in comparison with healthy controls. Individually, three kickboxers showed high *BP*_nd_ values in widespread brain regions, outside the range typically reported in healthy subjects, both in the current study and in previous studies [44]. Despite negative results on a group level, these findings seem to confirm that a degree of neuroinflammation may be present in currently active athletes without relationship to a recent (within three months) mild TBI. Thus, the widespread increased TSPO PET signal in these individuals could reflect the early consequences of RHI incurred during their career. The current study adds important information to the very limited amount of in vivo neuroimaging studies available targeted at microglial activation in contact sports athletes and is the first of its kind conducted in subjects participating in other sports than American football.

Previously, Coughlin and colleagues reported increased TSPO PET signal in multiple brain regions in currently active and recently retired NFL players, especially in the supramarginal gyrus [24], which was positively correlated with years of play. A similar relationship has been established in pathology studies, with a study in American football players (*n* = 66) reporting an association between the level of CD68 positivity and the number of years of playing football [47]. Years of play has also been linked with the risk of developing CTE, with an estimated doubling of risk after every 2.6 years of play, and increased NFT burden [48]. In our study, however, the upregulation of TSPO—as measured by the increased *BP*_nd_s—could not be correlated to the number of years training, training frequency, number of bouts fought and lost, or fighting style. On a group level, kickboxers showed superior performance on reaction tests evaluating basal mental speed. Previously, various psychological skills have been associated with increased performance in kickboxing, including relaxation and controlling anxiety, as well as personal characteristics such as mental toughness [49]. The 3 kickboxers with high *BP*_nd_s in widespread regions of the brain obtained scores within the 5th and 95th percentile on each test. Coughlin and colleagues similarly did not report significant differences in the performance on neuropsychiatric tests between athletes and controls despite evidence of TSPO upregulation [24]. This could be related to the relatively young age of the subjects included, as some studies indicated that aging is associated with increased prevalence of MCI and neurodegenerative disease in former contact athletes [50].

Evidence of activated microglia in athletes exposed to RHI has been less consistently reported in studies using CSF measurements. A previous study evaluating the levels of sTREM2 (a marker of microglia activation) in the CSF failed to find significant differences between symptomatic former NFL players (*n* = 68) and controls [19]. In the same study, a positive correlation between sTREM2 and p-tau was seen, which led to authors to suggest that re-activation of microglia may occur in the setting of neurodegeneration. The activation of microglia as measured with PET in a subset of kickboxers in our study may reflect a preclinical state associated with the development of cognitive, affective, and behavioral changes later in life, but this potential relationship remains to be fully elucidated. Future studies using TSPO PET may benefit from a longitudinal design to investigate the course of TSPO upregulation in athletes and a potential relationship with the onset of neuropsychiatric symptoms.

In our study, three boxers showed high *BP*_nd_ values in nearly all regions evaluated. The pattern of diffuse neuroinflammation is in accordance with the neuronal deficits detected using functional imaging techniques such as [^18^F]FDG PET, as a measure of brain metabolism, and perfusion SPECT in widespread areas of the brain. Provenzano et al. observed decreased metabolism in the posterior cingulate cortex, parieto-occipital, frontal lobes, and cerebellum in boxers (mean age 30) [51]. Amen and colleagues reported perfusion deficits in prefrontal, temporal, parietal, and occipital lobes and cerebellar regions in American football players (mean age 52) [52]. Interestingly in our study, kickboxers showed *increased* whole brain *K*_*1*_ values, which is indicative of relatively enhanced perfusion. Although speculative, this may be due to the lack of control for physical exercise in the control group. Previously, exercise has been associated with increased resting state cerebral perfusion in multiple areas of the brain in athletes [53]. Related to TBI, widespread areas of increased [^11^C]-PK11195 have also been reported, including areas unaffected on structural MRI [21].

Significantly increased FA values in the superor corona radiata were observed in kickboxers in comparison to controls. DTI measurements in other regions did not differ significantly between groups. Typical features of the microstructural damage previously described in professional boxers included reduced FA and increased MD in vulnerable brain regions in the white matter [54, 55], although negative results have also been reported [56]. Abnormally high FA values in the superior corona radiata were previously described in the context of mild traumatic brain injury by Mayer et al., who attributed this finding to cytotoxic edema or changes in water content within the myelin sheath [57]. A methodological drawback of some of the previous DTI reports is the manual drawing of VOIs, which we tried to overcome using an automatic atlas based approach. The lack of typical positive findings in our study may be a consequence of the inclusion of asymptomatic kickboxers and relatively young age.

### Limitations

In this study, years training, bouts fought, bouts lost, and training frequency were used as a surrogate marker of exposure to repetitive head injury. This is however not a perfect estimate, and individual variation, e.g., training intensity*,* may have resulted in undetected differences related to exposure. In addition, all subjects included were without any clinical complaints and relatively young, which may have reduced the a priori chances of increased TSPO binding. Second, [^11^C]-PK11195 is a first-generation TSPO PET tracer, which may lack sensitivity in comparison to newer ligands. On the other hand, [^11^C]-PK11195 has not been linked to genetically prone differences in binding affinity, an issue which requires genotyping when using second generation TSPO tracers [58]. Finally, the study was cross-sectional; therefore, longitudinal information related to TSPO binding and the potential onset of complaints is lacking.

## Conclusion

The current study fails to provide evidence of TSPO upregulation in currently active kickboxers on a group level, but the widespread increased binding of [^11^C]-PK11195 in approximately 1 out of 4 kickboxers suggests that neuroinflammation may be present as a consequence of repetitive head injury. In this group of asymptomatic kickboxers, this upregulation could not be correlated with the results on neuropsychological testing or DTI MRI findings. Future studies using a longitudinal design may determine whether the observed TSPO upregulation in active athletes is related to the development of neuropsychiatric symptoms.

## Supplementary Information

Below is the link to the electronic supplementary material.

Supplementary file1 (DOCX 93.2 KB)
