# Supplementary material for: Imaging of neuroinflammation due to repetitive head injury in currently active kickboxers
Source: Eur J Nucl Med Mol Imaging. 2022 Feb 15;49(9):3162–72. doi: 10.1007/s00259-022-05715-x (PMC9250484; doi:10.1007/s00259-022-05715-x)
Supplement: Supplementary file 1 — Supplementary file1 (DOCX 93.2 KB) [file 259_2022_5715_MOESM1_ESM.docx]

**Supplemental Data**

**TABLE 4.** Mean *K_1_* and *K_1_*/*K_2_* values of [^11^C]- PK11195 PET in kickboxers and controls

| **Brain region** | **Kickboxers (n=11)** | **Controls (n=11)** | **P value (Mann-Whitney U)** |
| --- | --- | --- | --- |
| **Brainstem *K_1_*** | 0.080 (0.0073) | 0.055 (0.0021) | 0.008 |
| **Frontal lobes *K_1_*** | 0.111 (0.015) | 0.071 (0.0043) | 0.004 |
| **Hippocampus *K_1_*** | 0.084 (0.014) | 0.058 (0.0029) | 0.061 |
| **Amygdala *K_1_*** | 0.089 (0.023) | 0.047 (0.0023) | 0.003 |
| **Temporal lobes *K_1_*** | 0.086 (0.0076) | 0.065 (0.0033) | 0.01 |
| **Parietal lobes *K_1_*** | 0.093 (0.0081) | 0.072 (0.0037) | 0.01 |
| **Occipital lobes *K_1_*** | 0.113 (0.012) | 0.083 (0.004) | 0.02 |
| **Insula and cingulate gyri *K_1_*** | 0.112 (0.013) | 0.074 (0.0036) | 0.002 |
| **Thalamus *K_1_*** | 0.106 (0.010) | 0.080 (0.0036) | 0.082 |
| **Striatum *K_1_*** | 0.102 (0.016) | 0.072 (0.0065) | 0.30 |
| **Cerebellum *K_1_*** | 0.119 (0.014) | 0.083 (0.0053) | 0.01 |
| **White matter *K_1_*** | 0.060 (0.0049) | 0.049 (0.0031) | 0.082 |
| **Whole brain *K_1_*** | 0.106 (0.0137) | 0.072 (0.0039) | 0.01 |
| **Whole brain *K_1_*/*K_2_*** | 0.52 (0.07) | 0.48 (0.02) | 0.82 |

**
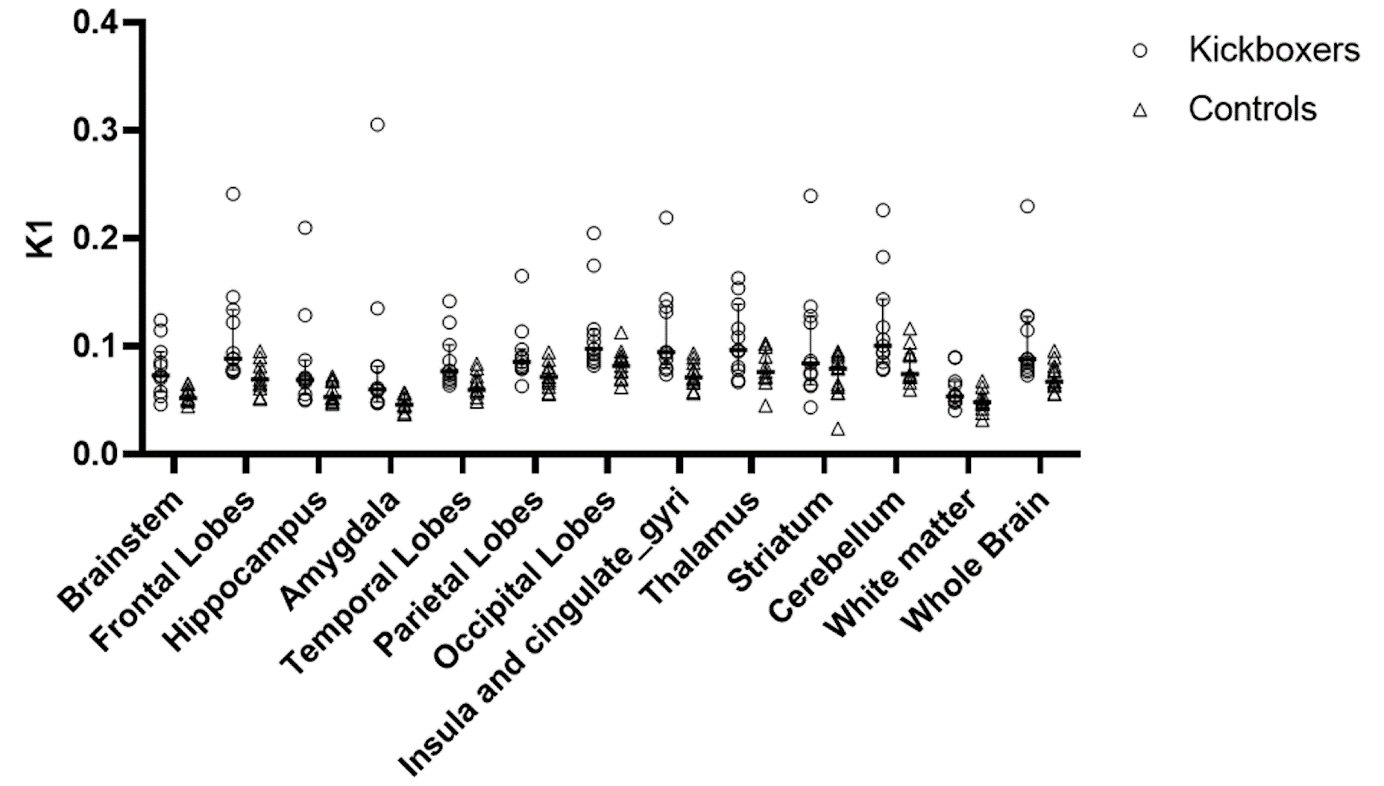
**

**FIGURE 5.** *K_1_* values of individual kickboxers and controls in several brain regions. The symbols represent individual subjects. The horizontal bar reflects the median and the error bars the 1^st^ and 3^rd^ quartile.

**TABLE 5** Median FA and MD values (interquartile ranges)in kickboxers and controls.

| **Brain region** | **Kickboxers (n=11)** | **Controls (n=11)** | **P value (Mann-Whitney U)** | **Effect size r** |
| --- | --- | --- | --- | --- |
| GCC FA  MD (x10^-3^) | 0.53 (0.51-0.56)  1.11 (0.97-1.12) | 0.52 (0.47-0.58)  1.02 (0.92-1.15) | 0.76  0.61 | 0.11  0.17 |
| BCC FA  MD (x10^-3^) | 0.52 (0.49-0.52)  0.80 (0.78-0.84) | 0.51 (0.49-0.53)  0.81 (0.78-0.83) | 0.71  0.92 | 0.13  0.04 |
| SCC FA  MD (x10^-3^) | 0.56 (0.54-0.57)  0.82 (0.78-0.88) | 0.54 (0.50-0.52)  0,85 (0.81-0.90) | 0.39  0.25 | 0.28  0.36 |
| ALIC R FA  MD (x10^-3^) | 0.53 (0.50-0.54)  0,70 (0.68-0.72) | 0.50 (0.49-0.52)  0.69 (0.67-0.71) | 0.31  0.43 | 0.32  0.24 |
| ALIC L FA  MD (x10^-3^) | 0.56 (0.53-0.58)  0.68 (0.62-0.70) | 0.55 (0.49-0.57)  0.64 (0.64-0.66) | 0.22  0.25 | 0.38  0.36 |
| PLIC R FA  MD (x10^-3^) | 0.59 (0.56-0.60)  0.73 (0.72-0.76) | 0.57 (0.56-0.59)  0.72 (0.70-0.75) | 0.20  0.35 | 0.40  0.29 |
| PLIC L FA  MD (x10^-3^) | 0.55 (0.51-0.56)  0.69 (0.67-0.71) | 0.53 (0.50-0.55)  0.68 (0.67-0.70) | 0.09  0.61 | 0.53  0.17 |
| RIC R FA  MD (x10^-3^) | 0.44 (0.42-0.48)  0.78 (0.75-0.80)) | 0.44 (0.38-0.48)  0.79 (0.77-0.80) | 0.61  0.47 | 0.17  0.30 |
| RIC L FA  MD (x10^-3^) | 0.52 (0.50-0.55)  0.82 (0,78-0.85) | 0.53 (0.52-0.54)  0.79 (0.75-0.80) | 0.47  0.04 | 0.23  0.64 |
| ACR R FA  MD (x10^-3^) | 0.37 (0.36-0.40)  0.76 (0.75-0.78) | 0.38 (0.35-0.39)  0.76 (0,74.0,79) | 0.61  0.86 | 0.17  0.06 |
| ACR L FA  MD (x10^-3^) | 0.39 (0.37-0.41)  0.79 (0.76-0.83) | 0.39 (0.39-0.40)  0.77 (0.75-0.81) | 0.22  0.39 | 0.38  0.27 |
| SCR R FA  MD (x10^-3^) | 0.42 (0.41-0.45)  0.71 (0.70-0.73) | 0.39 (0.39-0.40)  0.70 (0.69-0.74) | 0.002  0.31 | 0.89  0.32 |
| SCR L FA  MD (x10^-3^) | 0.44 (0.42-0.44)  0.72 (0.70-0.74) | 0.41 (0.39-0.42)  0.70 (0.69-0.73) | 0.006  0.11 | 0.83  0.49 |
| PCR R FA  MD (x10^-3^) | 0.39 (0.38-0.40)  0.76 (0.75-0.78) | 0.39 (0.38-0.40)  0.74 (0.72-0.77) | 0.31  0.22 | 0.32  0.37 |
| PCR L FA  MD (x10^-3^) | 0.40 (0.39-0.41)  0.80 (0.78-0.82) | 0.41 (0.40-0.43)  0.79 (0.77-0.82) | 0.39  0.35 | 0.28  0.30 |
| PTR R FA  MD (x10^-3^) | 0.50 (0.48-0.51)  0.76 (0.74-0.78) | 0.50 (0.48-0.54)  0.75 (0.73-0.76) | 0.81  0.43 | 0.09  0.26 |
| PTR L FA  MD (x10^-3^) | 0.50 (0.46-0.50)  0.86 (0.80-0.90) | 0.51 (0.50-0.53)  0.79 (0.77-0.83) | 0.06  0.10 | 0.57  0.51 |
| SLF R FA  MD (x10^-3^) | 0.42 (0.41-0.43)  0.71 (0.70-0.73) | 0.42 (0.39-0.43)  0.71 (0.70-0.74) | 0.71  0.47 | 0.13  0.22 |
| SLF L FA  MD (x10^-3^) | 0.44 (0.42-0.45)  0.72 (0.71-0.74) | 0.43 (0.41-0.44)  0.72 (0.70-0.73) | 0.20  0.65 | 0.40  0.14 |

**GCC** Genu corpus callosum, **BCC** Body corpus callosum, **SCC** Splenium corpus callosum, **ALIC R** Anterior limb of internal capsule right, **ALIC L** Anterior limb of internal capsule left, **PLIC R** Posterior limb of internal capsule right, **PLIC L** Posterior limb of internal capsule left, **RIC R** Retrolenticular part of internal capsule right, **RIC L** Retrolenticular part of internal capsule left, **ACR R** Anterior corona radiata right, **ACR L** Anterior corona radiata left, **SCR R** Superior corona radiata right, **SCR L** Superior corona radiata left, **PCR R** Posterior corona radiata right, **PCR L** Posterior corona radiata left, **PTL R** Posterior thalamic radiation right, **PTR L** Posterior thalamic radiation left, **SLF** **R** Superior longitudinal fasciculus right, **SLF** **L** Superior longitudinal fasciculus left
